# An Angle-Independent Multi-Color Display Electro-Responsive Hydrogel Film

**DOI:** 10.3390/gels9070568

**Published:** 2023-07-12

**Authors:** Huan Jiang, Yujiao Li, Fangfang Liu, Liping Sheng, Cheng-an Tao, Jianfang Wang

**Affiliations:** 1 College of Chemistry and Chemical Engineering, Hunan Normal University, Changsha 410081, China; 2 College of Science, National University of Defense Technology, Changsha 410073, China

**Keywords:** electro-responsive, hydrogel, photonic crystal, inverse opal, structural color

## Abstract

In nature, some organisms have the ability to camouflage to adapt to environmental changes; they blend with the environment by changing their skin colors. Such a phenomenon is of great significance for the research of adaptive camouflage materials. In this study, we propose a novel design scheme for the study of angle-independent photonic materials and successfully prepare an electrically tunable multi-color display angle-independent inverse opal photonic gel (IOPG). After photopolymerization of hydroxyethyl methacrylate with ionizable monomer acrylic acid (AA) in a long-range disordered opal template and etching, the angle-independent inverse opal photonic gel is obtained, presenting a single structural color. The electrically responsive color changes can be achieved at different angles. The color of the disordered AA-IOPG changes from green to blue-green when applying +4 V bias voltage and from green to orange when applying −4 V bias voltage. The electrochromism of the disordered AA-IOPG is mainly due to the local pH change caused by water electrolysis under bias voltage, which leads to a change of the swelling ratio. The disordered AA-IOPG shows high color tunability and durability through repeated opposite bias voltage tests, indicating that it is a promising conductive photonic material.

## 1. Introduction

Structural color is generated from the interaction between incident light and the periodic structure of photonic crystals. According to Bragg diffraction, the wavelength of structural color is related to the effective refractive index, the lattice parameter, and the diffraction angle [1,2,3]. The angle dependence of structural colors is important for some applications, such as in aesthetics and decoration and anti-counterfeiting [4]. However, angle-dependence is restricted in its application in fields, such as display and military stealth, that require color to remain unchanged over a large range of viewing angles. Therefore, the preparation of quasi-amorphous photonic crystal materials is an important way to broaden their application [5,6]. Harun-Ur-Rashid et al. fabricated SiO_2_ amorphous arrays, and its structural color essentially remained unchanged from 0° to 40°, which shows angle-independence [7]. Meng et al. synthesized polystyrene (PS) non-iridescent structural color films with high hydrophobicity and good mechanical stability through chaotic convective co-assembly. The structural color remained essentially unchanged in observation angle ranges of 0°–60°. With the help of carbon black, the color saturation was enhanced due to light absorption characteristic [8].

Responsive photonic crystals can respond to external stimuli through the visible changes of structural color [9,10]. They are widely used in color displays, ink-free rewritable paper [11,12], anti-military camouflage [13], information security [14,15] and biochemical sensors [16,17]. Electrically tunable photonic crystals are obtained through constructing electrochromic materials into photonic crystals, which can respond to external electric field stimulation through structural color changes due to volume expansion or contraction. Qu et al. obtained a lamellar PANI/PS-b-P2VP thin film which can provide an ideal multicolored electrochromic platform via spin-coating. The structural colors are widely controlled under a small electrical bias via the reversible change in the composition and domain spacing [18]. Yue et al. fabricated one-dimensional photonic crystals formed by ultrahigh-water-content polyelectrolyte layered hydrogels, and its structural colors are tunable to versatile colors when an electric field is applied [19]. Lee et al. synthesized an electrically tunable inverse opal photonic gel, and the structural colors are angle-dependent [20]. Although electrically tunable photonic crystals have been widely studied, there are few reports on angle-independent inverse opal photonic crystals, which have great applicability prospects in display and military stealth.

In this study, we produced an electrically tunable multi-color display angle-independent inverse opal photonic gel (IOPG). The long-term disordered opal template was used as a template, which was obtained through the simple chaotic convective flow assembly method. The precursor consisted of hydroxyethyl methacrylate (HEMA), and ionizable monomer acrylic acid (AA) was photopolymerized with the help of a “sandwich” structure of the template. After etching, the angle-independent inverse opal photonic gel (AA-IOPG) was obtained, and it can respond to electrical stimulation through changes in structural colors. The disordered AA-IOPG shows the characteristics of high color tunability and durability through repeated opposite bias voltage tests. We propose a novel design scheme for the study of angle-independent electrically tunable inverse opal photonic gel and achieve a tunable multi-color display. The AA-IOPG is a promising conductive photonic material and this work can lay the foundation for its application in the field of dynamic display.

## 2. Results and Discussion

A key procedure in the preparation of angle-independent inverse opal photonic gel film is the preparation of an angle-independent photonic crystal template. We adopt the method of horizontal growth micro-disturbance assembly, select the double-sided tissue paper composed of fiber and a porous surface (Figure S4) and overlay assembly to construct the template of an amorphous structure. Acrylic hydrogel electroactive material was further constructed based on the template. Under the action of applied voltage, the inverse opal micropore structure becomes larger or smaller, causing the hydrogel film to expand or contract and subsequently resulting in different angle-independent structural colors on the macro level.

**Figure 1:**
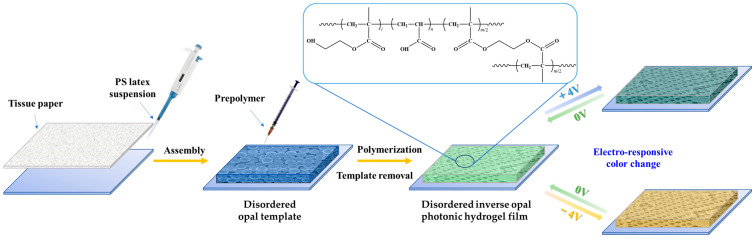
AA-IOPG constructed from 193 nm PS microspheres disordered template and its electrical response test diagram.

Figure 1 shows the specific preparation steps. Droplets of PS microsphere (193 nm in diameter) doped with trace CB were dropped on the glass substrate, and the surface of the suspension was quickly covered with a piece of treated double-sided tissue paper and heated to 70 °C to assemble. When the double-sided tissue paper was detached from the surface of the mixture, a blue disordered PS template could be obtained. The template was filled with the pre-polymer solution and placed under a UV lamp for photopolymerization. After soaking in tetrahydrofuran to remove PS microspheres, the green acrylic inverse opal photonic gel electroactive material was obtained. The ITO glass was used to construct a sandwich device. Since the light blue color of the ITO glass might affect the spectrum test, the upper layer of the ITO glass was replaced with acrylic glass attached to double-sided conductive copper foil in the actual test. By using physical pressing, the gel film was fixed on to the bottom ITO glass through the sealing film and connected to the positive electrode of the constant current power supply. In the phosphate electrolyte environment of 10^−3^ M and pH = 7, with the application of 4 V voltage, the structural color of the hydrogel film is slightly blueshifted, showing a blue-green macro color. With the application of −4 V voltage, the structural color of the hydrogel film is redshifted, and the macro color is orange-yellow.

**Figure 2:**
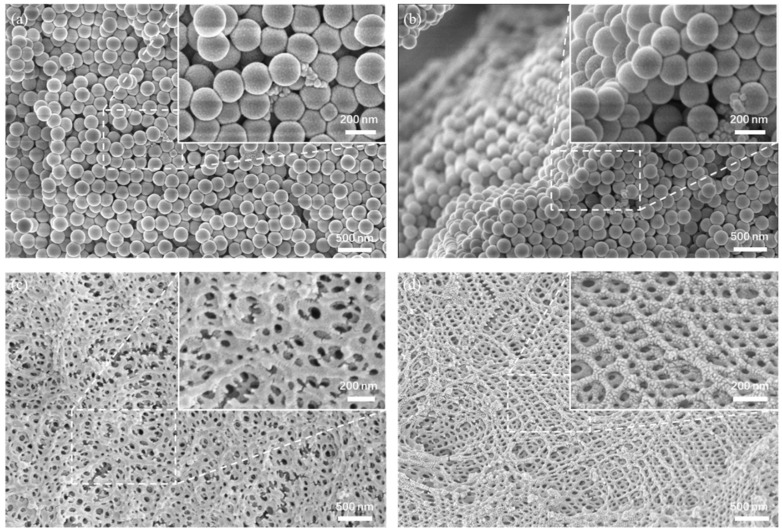
SEM images of disordered template constructed from 193 nm PS microspheres (doped with trace CB) (**a**) surface, (**b**) side face, and AA-IOPG (**c**) surface and (**d**) side face.

According to the SEM image, it can be seen that the assembled disordered template PS microsphere is uniform in size, with a calculated diameter of about 193 nm. The disordered template is a short-range ordered and long-range disordered structure, as shown in Figure 2a,b. Such a structure is spatially isotropic, and the refractive index difference inside is the same in all directions. When the size of the structural unit is consistent with the wavelength of visible light, a photonic band gap is generated. In the amorphous structure, the position of the photonic band gap is not affected by the direction of the incident light, so the structural color is angle-independent [21,22]. It can be seen that there are smaller conductive carbon black particles, which could absorb incident photons, reducing the propagation path of light in the template, thereby reducing the overall scattering intensity, making the scattering effect of the structural color wavelength become prominent and improving color saturation [23,24]. As shown in Figure 2c, the surface of the disordered AA-IOPG organic film contains a large number of irregular porous structures, including both large pores (~1 um) and small pores (~200 nm), and the large pores contain many small holes which were formed by the etching of the disordered template. The side face SEM images were obtained by cutting the sample, as shown in Figure 2d; the organic film has a disordered inverse opal structure inside, and the pore size of a single pore is about 200 nm. The thickness of the film is about 105 μm (Figure S5). Because of this disordered inverse opal structure, the organic film shows a single, angle-independent structural color on the macro level.

**Figure 3:**
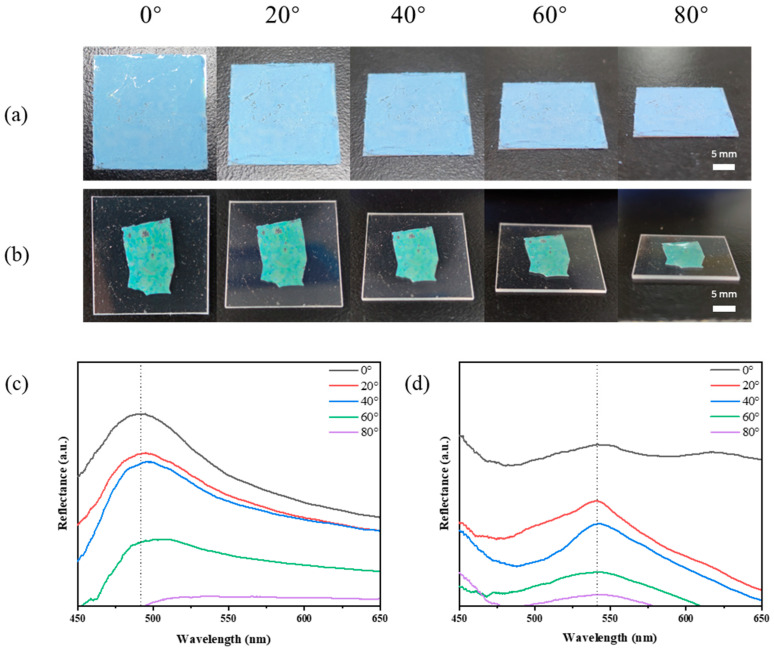
Optical photos (**a**,**b**) and reflective spectra (**c**,**d**) of disordered template constructed from 193 nm PS microspheres (**a**,**c**) and the corresponding AA-IOPG (**b**,**d**). The dotted lines in subfigure (**c**,**d**) is used to guide the sight.

In order to demonstrate the angle-independent property of the opal photonic crystal templates and the corresponding anti-opal photonic gels, the optical photos taken with digital cameras from different angles are observed. It can be seen that when the tilt angle is 80° and below, the structural color is almost unchanged (Figure 3a,b). The reflectance spectral data are in good agreement with the experimental results obtained from optical photographs (Figure 3c,d). Due to the limitation of experimental instruments, the diffuse reflection signals were achieved by changing the tilt angles of the probe, so when the test angle is large, the obtained spectral data reflectivity is very weak; that is because the probe can only receive limited light source signals. It can be shown that the opal photonic crystal templates and the corresponding anti-opal photonic gels are angle-independent.

**Figure 4:**
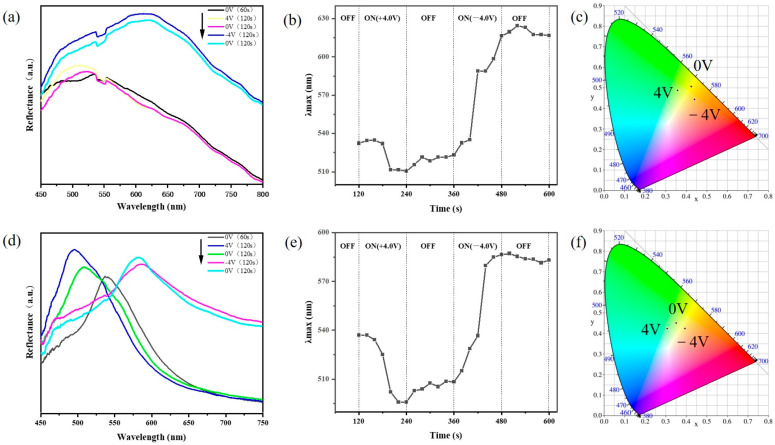
Spectra (**a**,**c**), peak wavelength (λ_max_, (**b**,**d**)) and chromaticity coordinates (**c**,**f**) of the disordered AA-IOPG under different voltages in (**a**–**c**) vertical direction (0°) and (**d**–**f**) tilt direction (12°). The black arrows in subfigure (**a**,**d**) indicate the order of the tests. The vertical bar in subfigure (**b**,**e**) represents the switching moment when the bias voltage is on and off. CIE data in (**c**): (0.424, 0.507) (0 V), (0.358, 0.488) (+4 V), (0.440, 0.444) (−4 V). CIE data in (**f**): (0.351, 0.451) (0 V), (0.307, 0.425), (0.394, 0.424) (−4 V).

**Figure 5:**
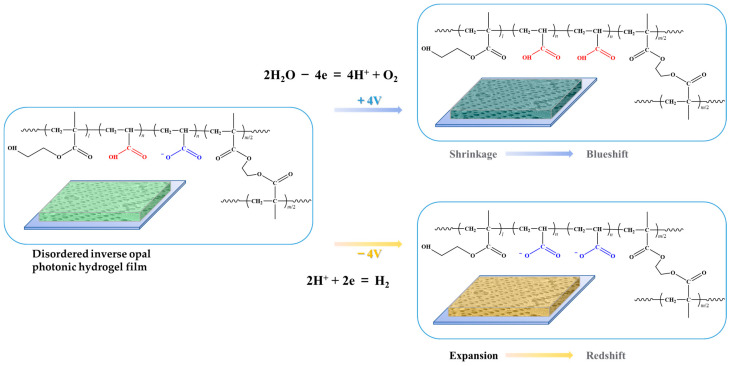
Schema of the macro structural color change mechanism on the molecular level when an electric field is applied.

**Figure 6:**
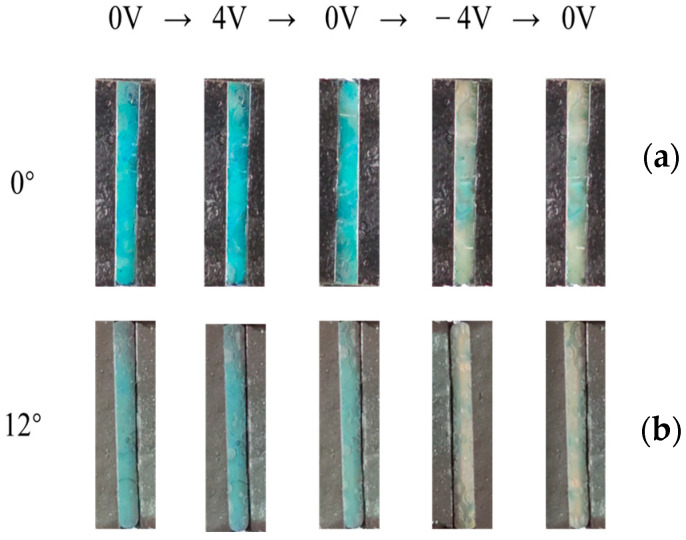
Optical photos of the disordered AA-IOPG under different voltages (**a**) in vertical direction (0°) and (**b**) tilt direction (12°).

Another crucial test is the electrical response of the disordered AA-IOPG. The variation of reflection spectra over time in the vertical direction (0°) and tilt 12° with ±4 V bias voltages are shown in Figure 4. The reflection spectral data in the vertical direction (0°) are shown in Figure 4a. At the beginning of the test, the voltage is adjusted to 0 V, and the circuit is turned on to form a closed loop for 120 s to eliminate the effect of the potential. After applying 4 V voltage for 120 s, the maximum reflective peak wavelength λ_max_ blueshifts from 540 nm to 510 nm (Figure 4b), and the macro structural color changes from green to blue-green. This is probably because H^+^ is generated from the water decomposing on the surface of the ITO conductive glass at the anode and the local pH value decreases, and then the—COO^−^ groups of the AA-IOPG tend to protonate to form—COOH and migrate to the anode, resulting in the decrease of pore size, lattice spacing d and λ_max_ of the gel film, so the structural color blueshifts (Figure 5), which is consistent with the previous report [20]. When the voltage is adjusted to 0 V for 120 s, the hydrogel recovers slowly, with a slight redshift, and the macroscopic structural color is essentially unchanged, mainly because the pH value of the environment where the AA-IOPG is essentially unchanged and the only recovery force depends on the elastic of the gel film itself. The color change is shown in Figure 6a.

Then the −4 V voltage is applied and maintained for 120 s. The maximum reflection peak is redshifted to 620 nm, and the macro structural color changes from green to orange-yellow because H^+^ on the surface of the ITO conductive glass makes electrons form H_2_ at the cathode. The rapid consumption of H^+^ leads to an increase in the local pH value, and the—COO^−^ group of the AA-IOPG repulse the cathode and migrate to the anode. As a result, the pore size, the lattice spacing d and λ_max_ of the gel film are enlarged, and the structural color is redshifted (Figure 5). When the voltage is adjusted to 0 V again and maintained for 120 s, the hydrogel recovers slowly, slightly blueshifts, and the macro structural color is still orange-yellow. The chromaticity coordinates of the AA-IOPG changed from CIE: (0.424, 0.507) under 0 V to (0.358, 0.488) under +4 V and to (0.440, 0.444) under −4 V in the vertical direction (0°), as shown in Figure 4c.

The variation of the reflection spectrum data results in the tilt direction (12°) is the same as that measured in the vertical direction, as shown in Figure 4d,e, and the corresponding optical photo changes are also consistent. As shown in Figure 6b, the macro structural color can change from green to blue-green and then to orange-yellow. The chromaticity coordinates of the AA-IOPG changed from CIE: (0.351, 0.451) under 0 V to (0.307, 0.425) under +4 V and to (0.394, 0.424) under −4 V in the tilt direction (12°), as shown in Figure 4f. The variation of the reflection spectra over time in other tilt angles are shown in Figure S6. These results indicate that acrylic hydrogels with an inverse opal structure can achieve angle-independent color conversion under electrical stimulation.

**Figure 7:**
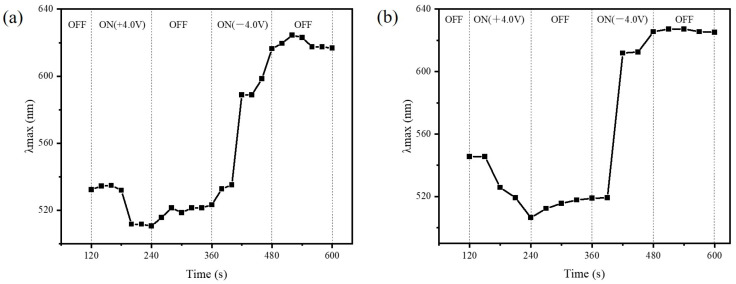
The peak wavelength changes of the disordered AA-IOPG under different voltages originally (**a**) and after six months (**b**). The vertical bar represents the switching moment when the bias voltage is on and off.

The acrylic inverse opal photonic gel film has good durability. Figure 7 shows the peak wavelength changes of the disordered AA-IOPG under different voltages originally and after six months. A test cycle starts from a positive voltage and turn-off voltage to a negative voltage and turn-off voltage. The corresponding spectral maximum reflectance peak is first blueshifted and then redshifted. After six months, the AA-IOPG film exhibits the same performance, proving its good durability (Figure S7). When the tested gel film is placed in a phosphate electrolyte of 10^−3^ M and pH = 7, the gel film can quickly return to its initial state. However, the ITO conductive glass used in the test would be oxidized at 4 V voltage, so the second cycle test should change the ITO glass to form another device using the same gel film. After several tests, the gel film still maintains good optical properties only if there is enough aqueous solution for supplying or consuming H^+^.

## 3. Conclusions

In this study, we constructed an angle-independent acrylic inverse opal photonic gel based on a disordered template assembled by PS emulsions doped with trace amounts of conductive carbon black. Optical photographs and reflection spectral data show that the structural color is almost unchanged at 80° and below. The electrical tunability of the IOPG in aqueous media was demonstrated at a low voltage (±4 V). HEMA and AA are photopolymerized into charged copolymers, and an IOPG with monochromatic reflection color is formed when PS microspheres are removed. In an environment of 10^−3^ M and pH = 7 PBS electrolyte, a blue shift is produced when applying positive voltage and a red shift is produced when applying the opposite negative voltage, and the initial state is recovered gradually under the turn-off voltage. In the visible spectrum range, the gel film can realize the red, green and blue multi-color reflection structural color display. The spectral test of the tilt angle shows that the electrotuned color change of the gel film is angle-independent. Repeated reverse bias cycle tests on the gel film confirm that the electrically driven color change is reversible, and the gel film has a good durability. Our research provides a new design scheme for angle-independent reflection multi-color display technology. The prepared gel films also have the advantages of wide color adjustability, low power consumption and good durability, and are expected to promote their application in display equipment, camouflage, anti-counterfeiting and other related fields, and could further be combined with optical, thermal and other functional materials to obtain multifunctional devices for storage, catalysis, sensing, and so on.

## 4. Materials and Methods

### 4.1. Materials

Styrene (GC ≥ 99.5%), potassium persulfate (KPS) and sodium dodecyl sulfate (SDS) were purchased from Sinopharm Chemical Reagent Co., Ltd., Shanghai, China, and used to synthesize polystyrene (PS) microspheres. Hydroxymethyl methacrylate (HEMA) purchased from Sigma-Aldrich, St. Louis, MO, USA; ethylene glycol dimethacrylate (EGDMA, GC = 98%) purchased from J&K Scientific, San Jose, CA, USA; and acrylic acid (AA, GC ≥ 98%) and Irgacure1 (photoinitiator, GC = 99%) purchased from Adamas-beta^®^ Inc., Shanghai, China, were used to prepare photonic gel monomers. Deionized (DI) water was prepared using a laboratory water purification system. Different amounts of Na_2_HPO_4_ (Sinopharm Group, Shanghai, China) and NaH_2_PO_4_ (Sinopharm Group) were dissolved in deionized water, and phosphate buffers with different pH values were calculated. Conductive carbon black (CB, ~23 nm) was purchased from Jiangsu XFNANO Materials Tech Co., Ltd., and was added in small amounts to enhance the color saturation of the template. A black long-tail ticket holder (metal clip) and black insulation tape were purchased from Zhejiang Ninhai Deli Group Co., Ltd., Ningbo, China; the sealing film PM-996 was purchased from Parafilm Co., Ltd., Warrington, PA, USA; the double-sided tissue paper was purchased from Dongguan Blue Sky Paper Co., Ltd., Dongguan, China; the methyl silicone oil was purchased from Longxu (Shanghai, China) Organosilicone Technology Co., Ltd. Acrylic transparent organic glass (transmittance ≥ 92%) was purchased from Jiangxi Oujia Industry Co., Ltd., Fuzhou, China; indium tin oxide (ITO) conductive glass (square resistance ≤ 6 Ω, transmittance ≥ 84%, film thickness 185 nm, light blue) and double-sided conductive copper foil were purchased from South China Xiangcheng Technology Co., Ltd., Shenzhen, China. ITO conductive glass was used after ultrasonic cleaning with acetone, deionized water and anhydrous ethanol. Tetrahydrofuran (GC ≥ 99.5%), acetonitrile (GC ≥ 99.5%) and ethanol (GC ≥ 99.7%) were purchased from Sinopsin Chemical Reagent Co., Ltd., Shanghai, China, and used directly after receiving them.

### 4.2. Preparation of Disordered Template from Monodisperse Polystyrene Microspheres

Monodisperse polystyrene microspheres were prepared by emulsion polymerization using styrene as a monomer, sodium dodecyl sulfate as an emulsifier and potassium persulfate as an initiator. The emulsion with differently sized microspheres was obtained by adjusting the amount of emulsifier, as shown in Table S1.

The specific method is as follows. A certain amount of sodium dodecyl sulfate and 135 mL deionized water was added to a 250 mL three-neck flask. After condensing in water and 300 r/min mechanical stirring for 15 min, 15 g styrene was added. After heating in an oil bath for 30 min at 85 °C, potassium persulfate (1 wt% of styrene) was added. The polystyrene (PS) microsphere emulsion was obtained after 5 h.

A total of 0.1 g conductive carbon black, 0.08 g SDS and 40 g DI Water were added into a beaker and mixed evenly by stirring and using ultrasonic waves to obtain a carbon black solution.

The monodisperse PS emulsion (PS particle concentration was 10 wt%) with different particle sizes was mixed with the carbon black solution (5 wt% of PS emulsion) and dispersed by ultrasonic waves for 30 min. The glass substrate was placed on a 70 °C heating plate, and an appropriate amount of the abovementioned mixture was dropped onto the glass substrate, and the treated double-sided tissue paper was immediately placed on the surface of the mixture. The double-sided tissue paper should be cut into the same size as the glass substrate before use, wiped with methyl silicone oil and dried. Through the pore evaporation guide of the two-sided tissue paper, the mixture particles underwent Brownian motion and assembled disorderly. When the double-sided tissue paper was removed from the surface of the mixture, the disordered PS template could be obtained. The SEM images and the optical photos of the templates are shown in Figures S1 and S2.

### 4.3. Preparation of Disordered Inverse Opal Hydrogel Film

The as-assembled PS template was covered with a clean acrylic glass sheet and held with metal clips to form a sandwich.

The monomer mixture was prepared by mixing 2.5 g hydroxyethyl methacrylate (HEMA) as a hydrogel building block, 0.100 g ethylene glycol dimethacrylate (EGDMA) as a crosslinking agent, 0.075 g Irgacure1 as a photoinitiator, and 0.0346 g AA (2.5 mol% of HEMA) and 0.0625 g DI water. After filling the PS opal template with the monomer mixture for 10 min, photopolymerization was performed using a UV lamp (100 W, spectral line) for 2 h. After disassembly, the sample was immersed in tetrahydrofuran and extracted by a Soxhlet extractor for 2 days to completely remove the PS template.

After the template was removed, the prepared acrylate inverse opal photonic gel (AA-IOPG) film was rinsed with chloroform, acetonitrile and deionized water and finally immersed in an aqueous buffer solution. The optical photos of the AA-IOPG are shown in Figure S3.

### 4.4. Fabrication of Sandwich Type Electrical Device

The acrylic glass and the ITO conductive glass were cut into a 2 cm × 2 cm size, and the ITO conductive glass was ultrasonically washed with acetone, DI water and ethanol. Double-sided conductive copper foil was adhered to the surface of the acrylic glass, leaving a square hole (6 mm × 12 mm) to facilitate the reflection spectrum test. The other side of the acrylic glass was covered with black insulation tape to isolate the influence of the copper foil’s color on the reflection spectrum test. The conductive copper foil surface of the acrylic glass was connected to the negative electrode of the constant current power supply as the cathode. The gel film was spread out and attached to the conductive layer of the bottom ITO conductive glass, which was connected to the positive electrode of the constant current power supply serving as the anode with double-sided conductive copper foil, and the sealing film was used to fix the gel film. A phosphate electrolyte of 10^−3^ M and pH = 7 was injected and the two electrodes were clamped with metal clips to form a sealed device. The sealing film acted as a spacer, and the spacing between the two electrodes was about 120 nm.

Continuous replenishment of the electrolyte to the device was required during the electrical structural colors tuning tests of the disordered AA-IOPG using a constant current power supply to apply a bias between the two electrodes.

### 4.5. Characterizations

The optical photos and videos were taken with a digital camera in professional mode. SEM images were taken with the Zeiss Sigma 300 SEM (Carl Zeiss AG, Oberkochen, Germany) and Hitachi Advanced Scanning Electron Microscope FlexSEM 1000 II (Hitachi Ltd., Tokyo, Japan), and all samples were sprayed with gold before testing. In addition, the disordered AA-IOPG gel film was treated with CO_2_ drying with the American Tousimis Samdri-PVT-3D (Tousimis Research Corporation, Rockville, MD, USA) critical point dryer before gold spraying. The applied voltage was provided by the constant current power supply of Nanjing NANDA Wanhe Technology Co., Ltd., Nanjing, China. The reflectance spectral data were provided by the USB2000+ FiberSpectrometer of the Marine Optical Miniature (Ocean Optics, Orlando, FL, USA), and the data for different angle tests were measured by tilting the probe and changing the angle to the normal of the sample.

## Supplementary Materials

The following supporting information can be downloaded at: https://www.mdpi.com/article/10.3390/gels9070568/s1, Table S1: emulsifier SDS dosage and PS particle size, Figure S1: SEM images of templates assembled from PS microspheres doped with trace conductive carbon black by micro disturbance of double-sided copy paper cover, Figure S2: Optical photos of templates assembled from different particle sizes PS microspheres doped with trace conductive carbon black by micro disturbance of double-sided copy paper cover, Figure S3: Optical photos of disordered AA-IOPG based on disordered templates assembled from different particle sizes PS microspheres doped with trace conductive carbon black by micro disturbance of double-sided copy paper cover (vertically and tilt 30°), Figure S4: SEM images of double-sided tissue paper, Figure S5: SEM image of disordered AA-IOPG organic film constructed based on 193 nm polystyrene microspheres (thickness of film ~105 μm), Figure S6: Variation of reflection spectra over time in the direction of vertical (a), tilt 12° (b), 25° (c) and 40° (d) with different bias voltages (±4.0 V), Figure S7: The reflection spectra over time in vertical direction of original (a) and after six months (b) of disordered AA-IOPG under different bias voltages (±4.0 V), Video S1: Color change of the disordered AA-IOPG film along with the bias voltages.
