# Supplementary material for: An Angle-Independent Multi-Color Display Electro-Responsive Hydrogel Film
Source: Gels. 2023 Jul 12;9(7):568. doi: 10.3390/gels9070568 (PMC10379048; doi:10.3390/gels9070568)
Supplement: Supplementary file 1 [file gels-09-00568-s001.zip › ESI-04-revised.pdf]

Supporting Information

An Angle-Independent Multi-Color Display Electro-Responsive Hydrogel Film

Huan Jiang [1,2,†], Yujiao Li [2,†], Fangfang Liu [2], Liping Sheng [1,*], Cheng-an Tao [2,*] and Jianfang Wang [2]

1 College of Chemistry and Chemical Engineering, Hunan Normal University, Changsha 410081, China
2 College of Science, National University of Defense Technology, Changsha 410073, China
* Correspondence: shenglp@hunnu.edu.cn (L.S.); taochengan@nudt.edu.cn (C.-a.T.)
† These authors contributed equally to this work.

Table S1. emulsifier SDS dosage and PS particle size

NO.	SDS dosage (mg)	particle size (nm)	Reflective peak wavelength (nm)
1	80.8	193	487.51
2	70	210	522.53
3	67.5	236	571.1
4	59.1	257	640.52

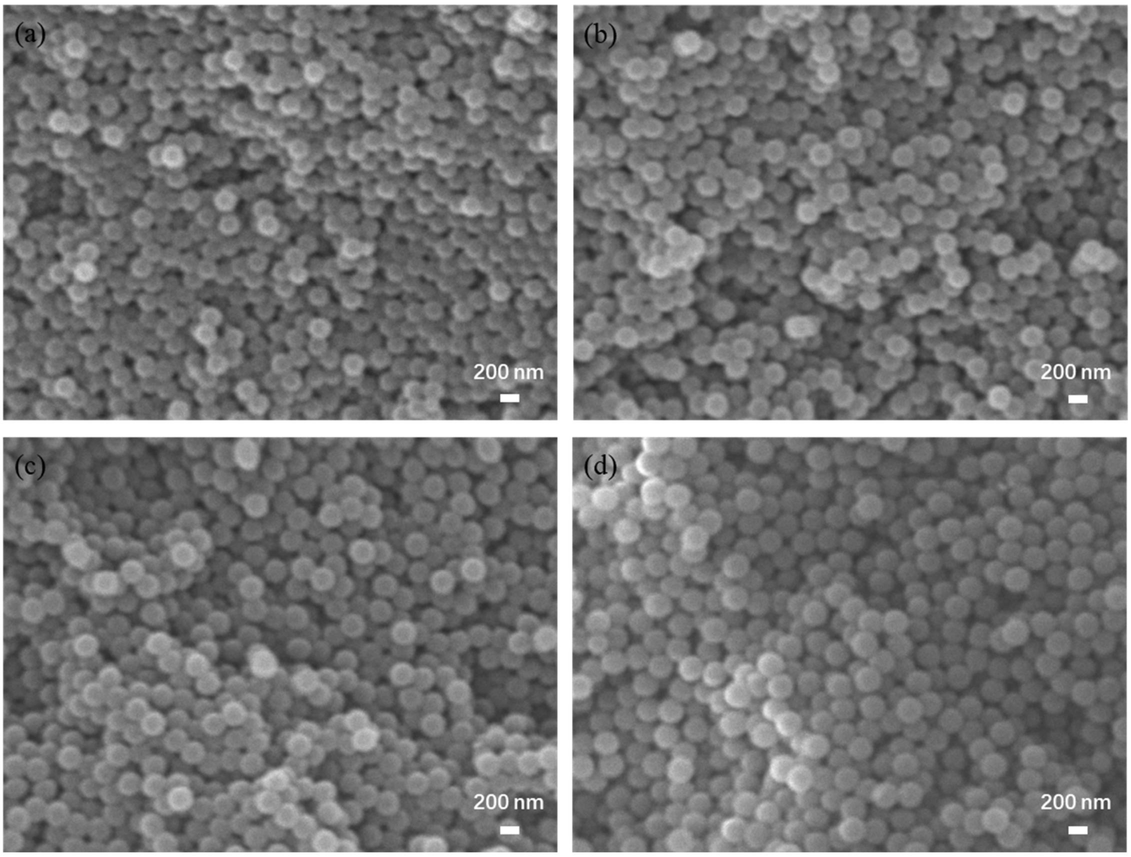

Figure S1. SEM images of templates assembled from PS microspheres doped with trace conductive carbon black by micro disturbance of double-sided copy paper cover. The particle sizes of the PS microspheres are (a) 193 nm, (b) 210 nm, (c) 236 nm and (d) 257 nm.

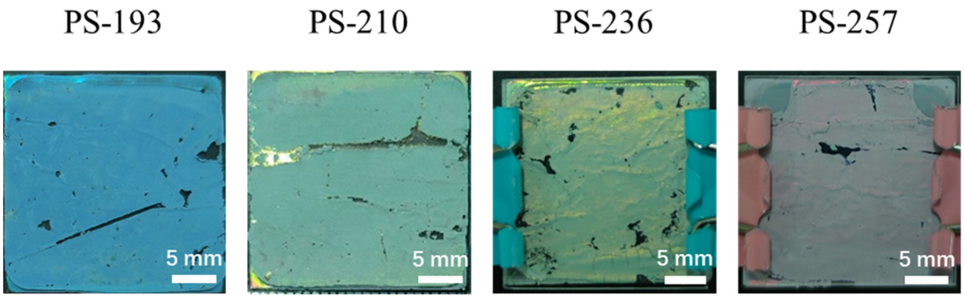

Figure S2. Optical photos of templates assembled from different particle sizes PS

microspheres doped with trace conductive carbon black by micro disturbance of double-sided copy paper cover.

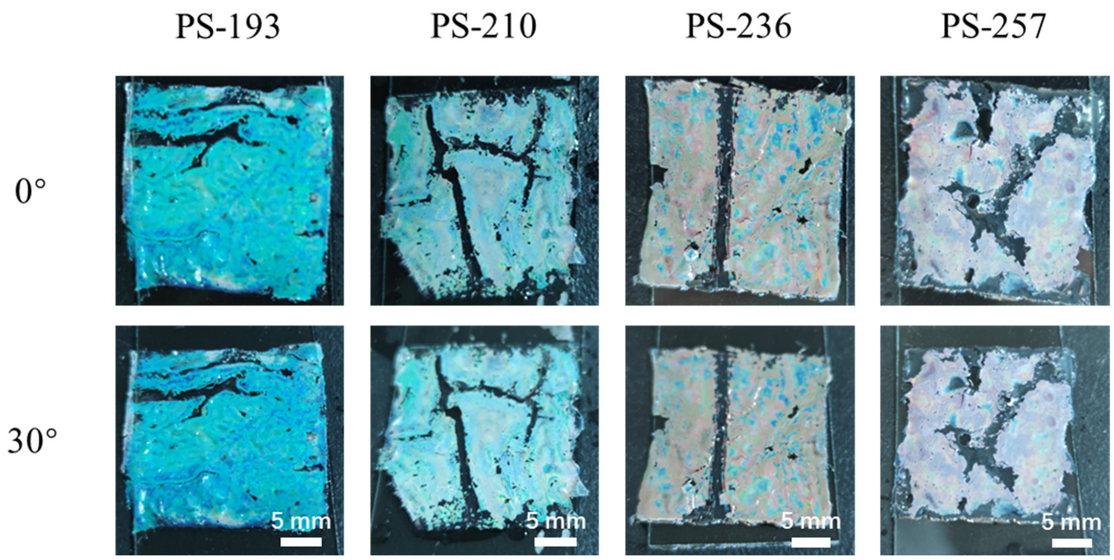

Figure S3. Optical photos of disordered AA-IOPG based on disordered templates assembled from different particle sizes PS microspheres doped with trace conductive carbon black by micro disturbance of double-sided copy paper cover (vertically and tilt 30°).

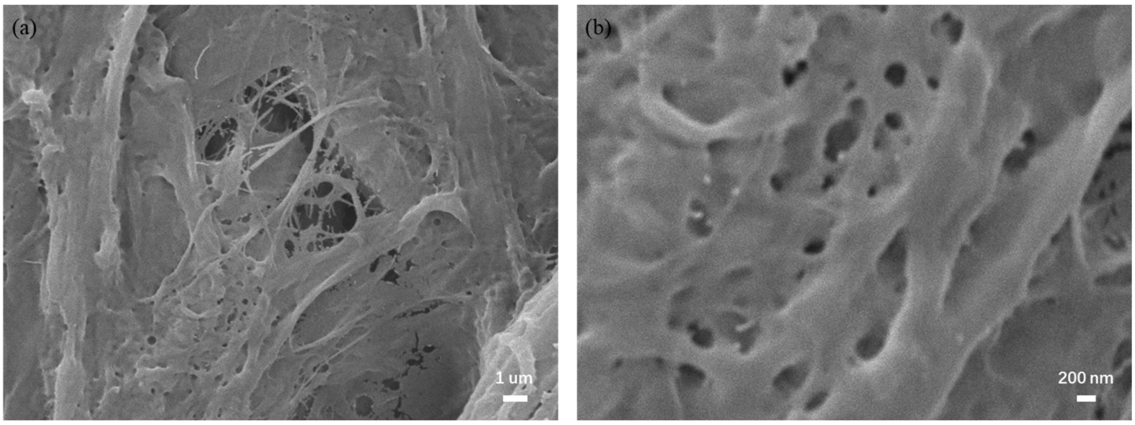

Figure S4. SEM images of double-sided tissue paper.

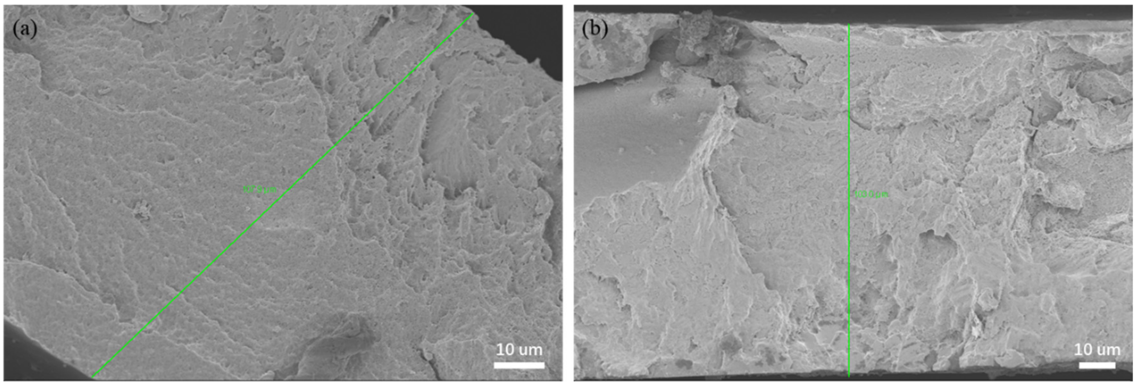

Figure S5. SEM image of disordered AA-IOPG organic film constructed based on 193 nm polystyrene microspheres (thickness of film ~105 μm).

(a)

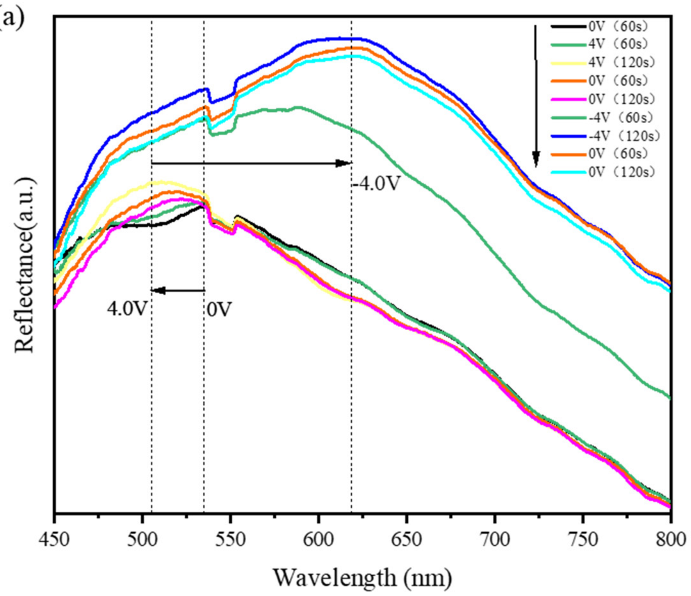
0V（60s）
4V（60s）
4V（120s）
0V（60s）
0V（120s）
-4V（60s）
-4V（120s）
0V（60s）
0V（120s）
-4.0V
4.0V
0V
Reflectance(a.u.)
450
500
550
600
650
700
750
800
Wavelength (nm)

(b)

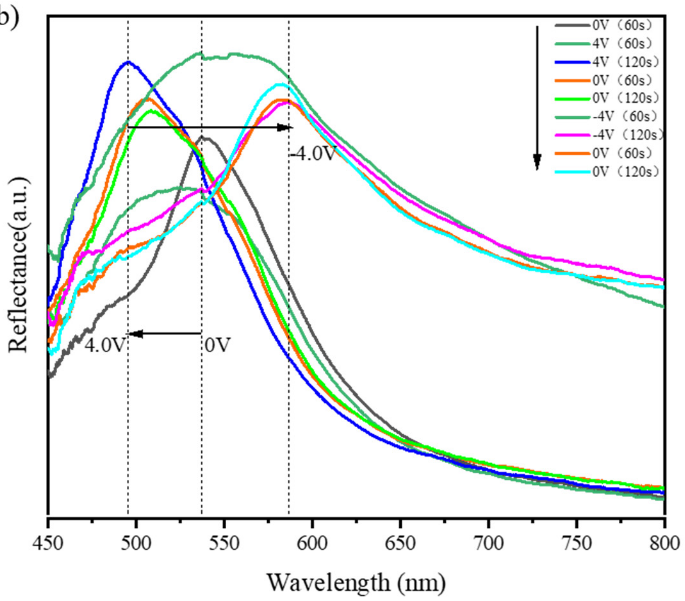
0V（60s）
4V（60s）
4V（120s）
0V（60s）
0V（120s）
-4V（60s）
-4V（120s）
0V（60s）
0V（120s）
-4.0V
4.0V
0V
Reflectance(a.u.)
450
500
550
600
650
700
750
800
Wavelength (nm)

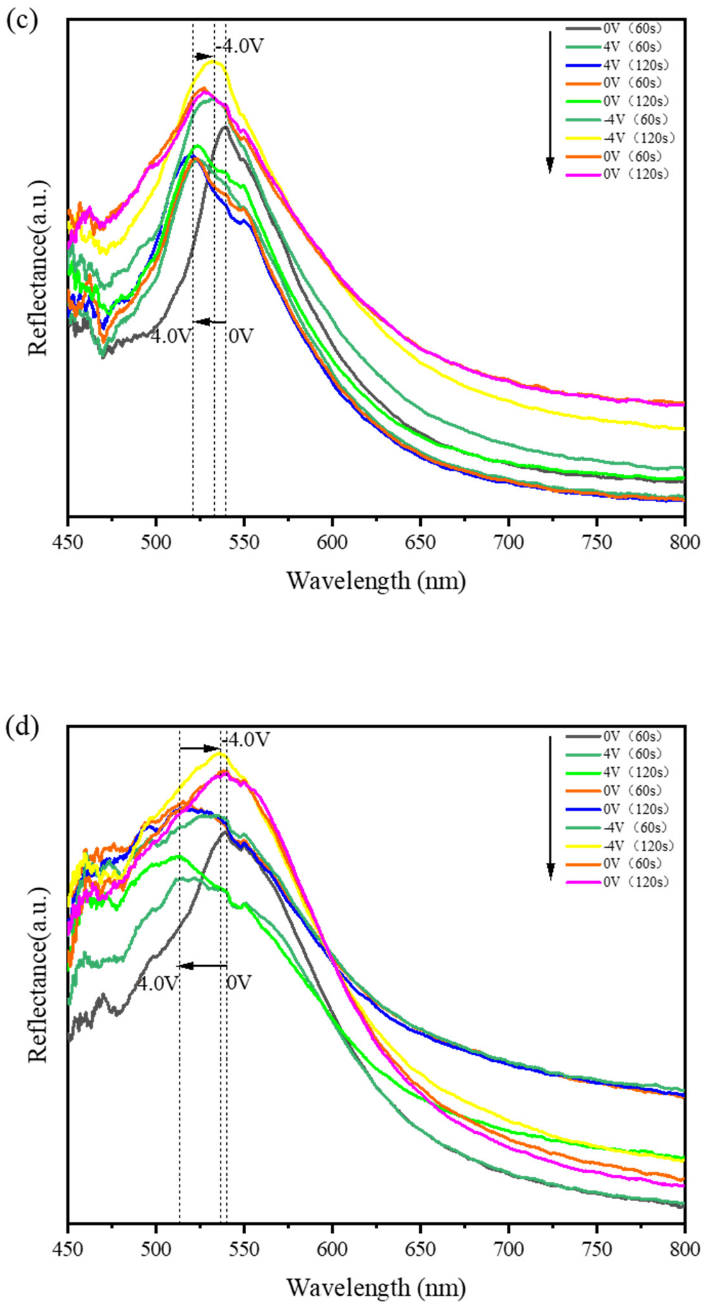

Figure S6. Variation of reflection spectra over time in the direction of vertical (a), tilt 12° (b), 25° (c) and 40° (d) with different bias voltages (± 4.0V).

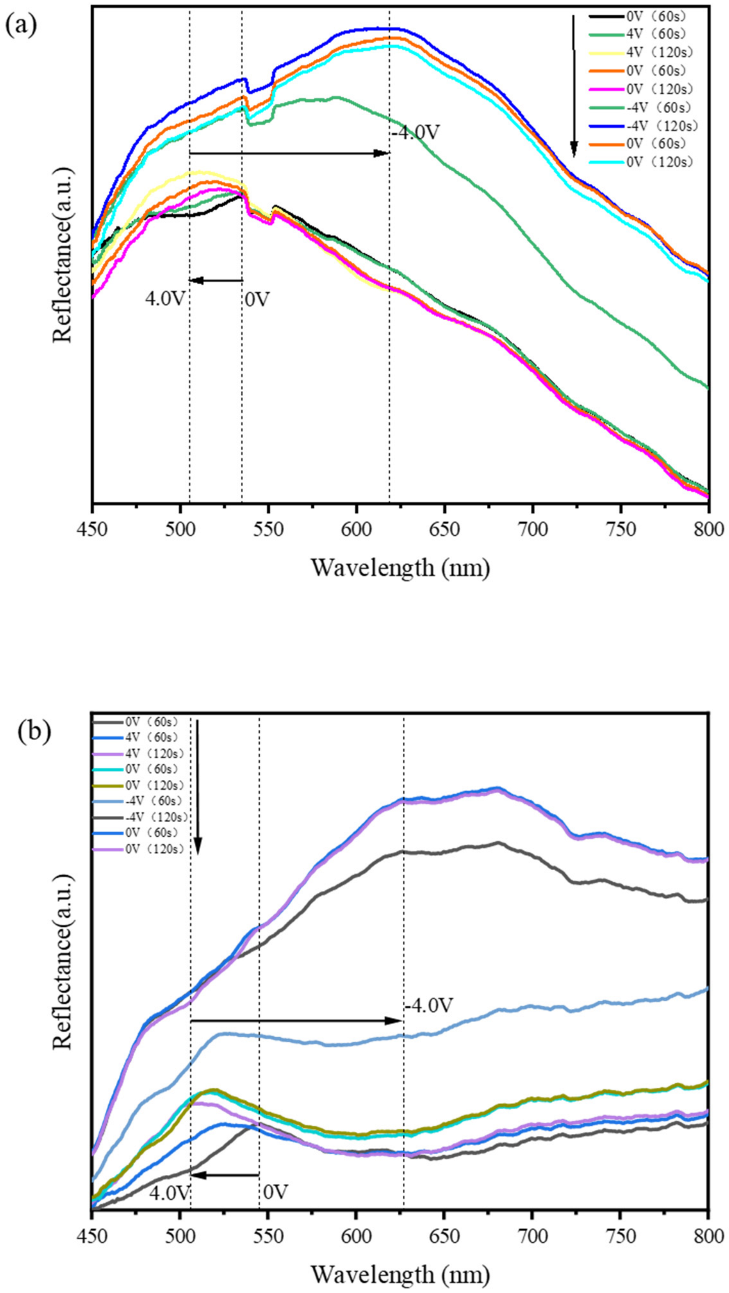

Figure S7. The reflection spectra over time in vertical direction of original (a) and after six months (b) of disordered AA-IOPG under different bias voltages (± 4.0V).
